# Supplementary material for: FOCUS: an alignment-free model to identify organisms in metagenomes using non-negative least squares
Source: PeerJ. 2014 Jun 5;2:e425. doi: 10.7717/peerj.425 (PMC4060023; doi:10.7717/peerj.425)
Supplement: Figures S1--S8 [file peerj-02-425-s005.doc]

**Supplementary Material**

For the SimShort simulated metagenome, we also compared FOCUS against PhymnBL, and RAIphy the tools default database in for other taxonomic levels (see Supplemental  Fig. 1 - 5). The same was done for the SimHC synthetic metagenome (see Supplemental  Fig. 6 - 8).

**
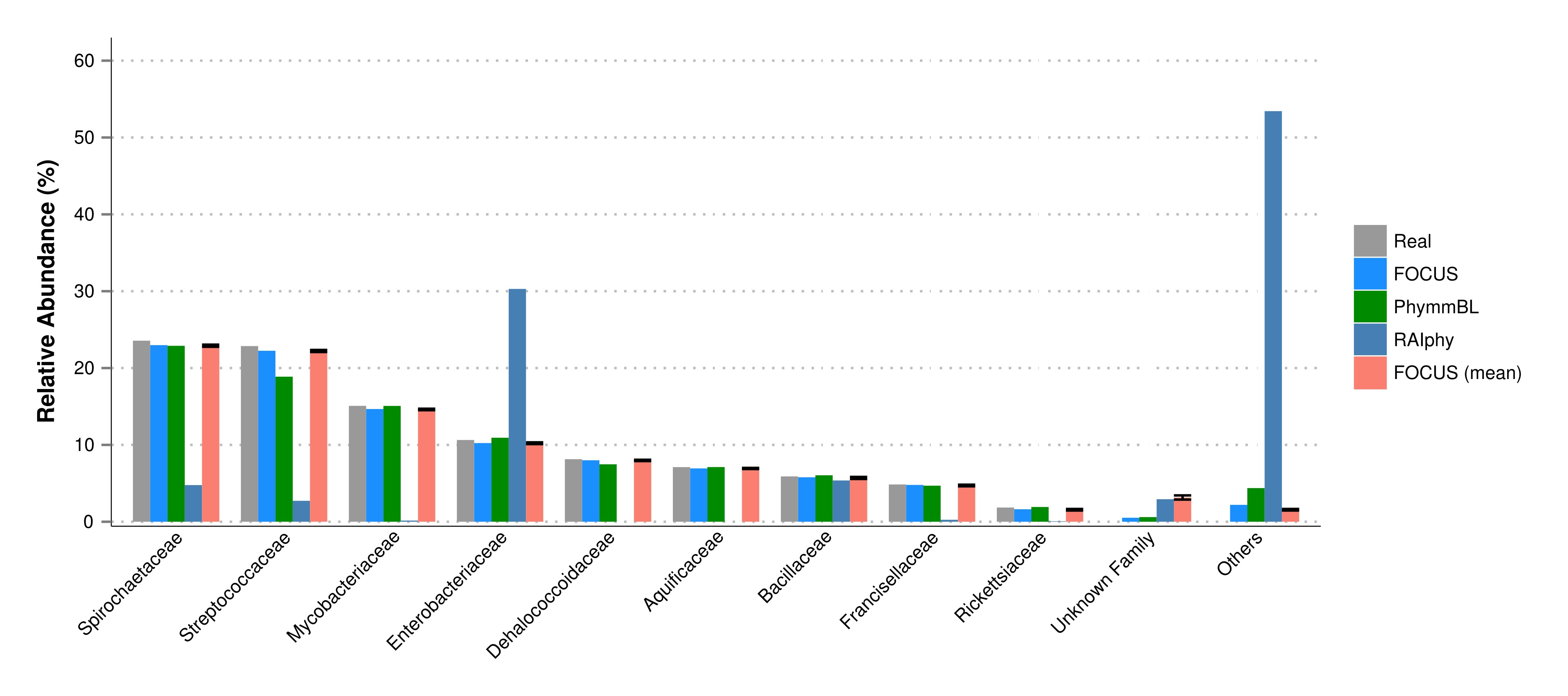
**

**Supplemental  Fig. 1.** Family-level taxonomy classification for the SimShort dataset using FOCUS, PhymnBL, RAIphy, and FOCUS (mean).


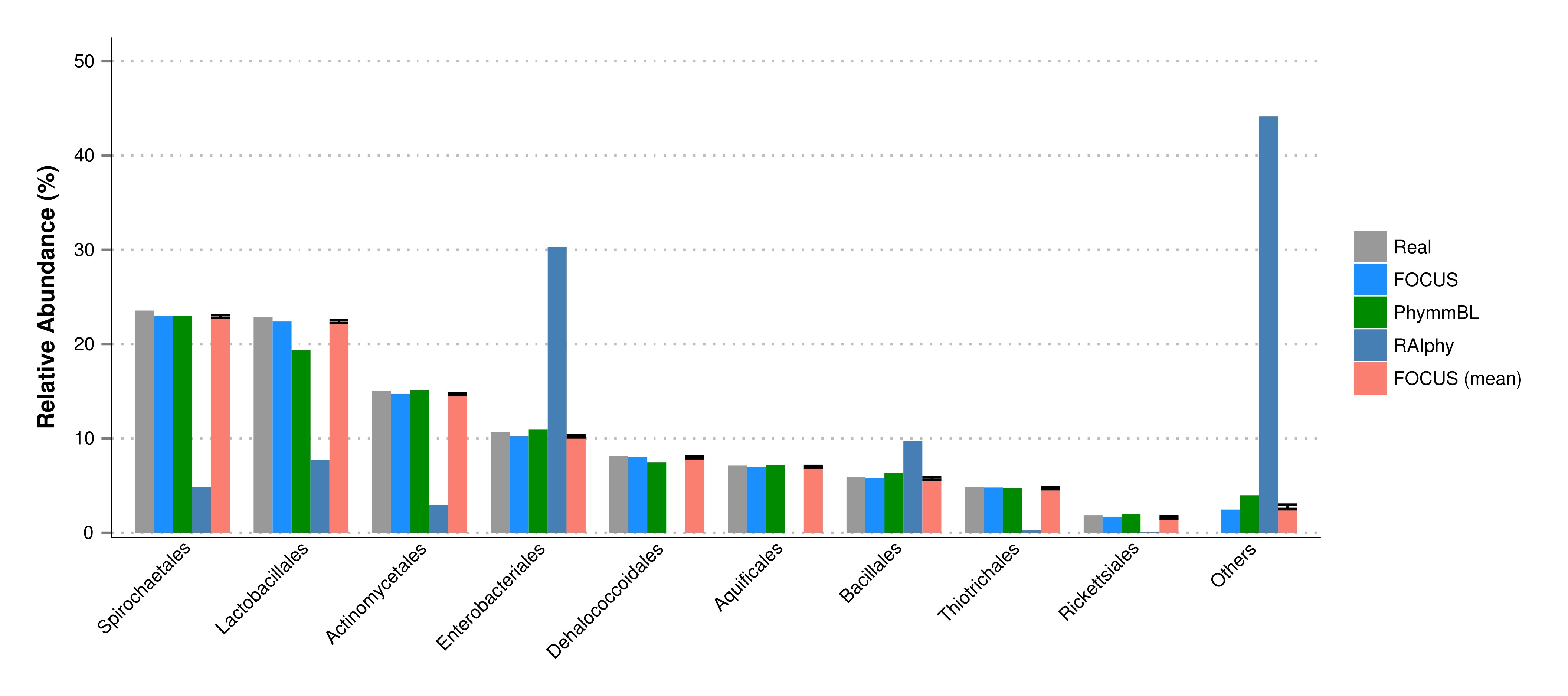


**Supplemental  Fig. 2.** Order-level taxonomy classification for the SimShort dataset using FOCUS, PhymnBL, RAIphy, and FOCUS (mean).


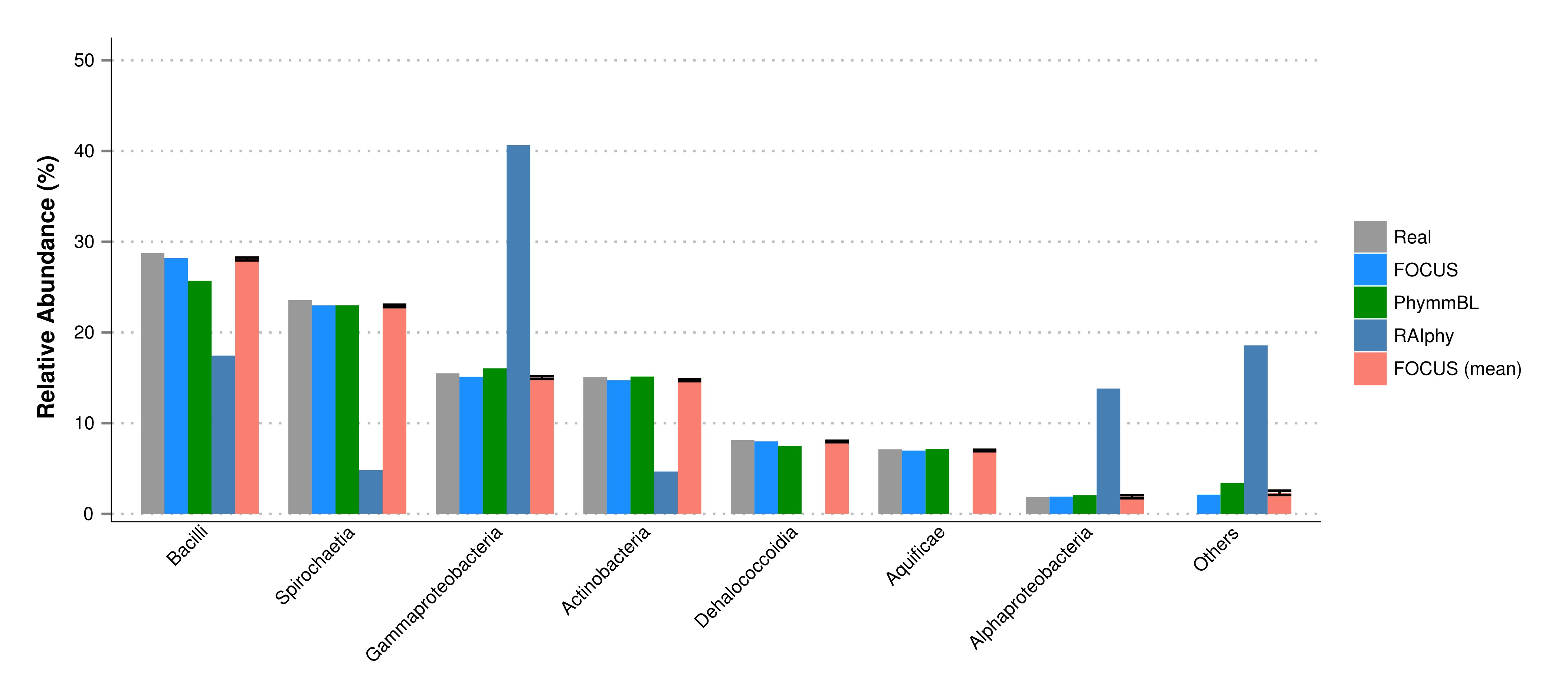


**Supplemental  Fig. 3.** Class-level taxonomy classification for the SimShort dataset using FOCUS, PhymnBL, RAIphy, and FOCUS (mean).

**
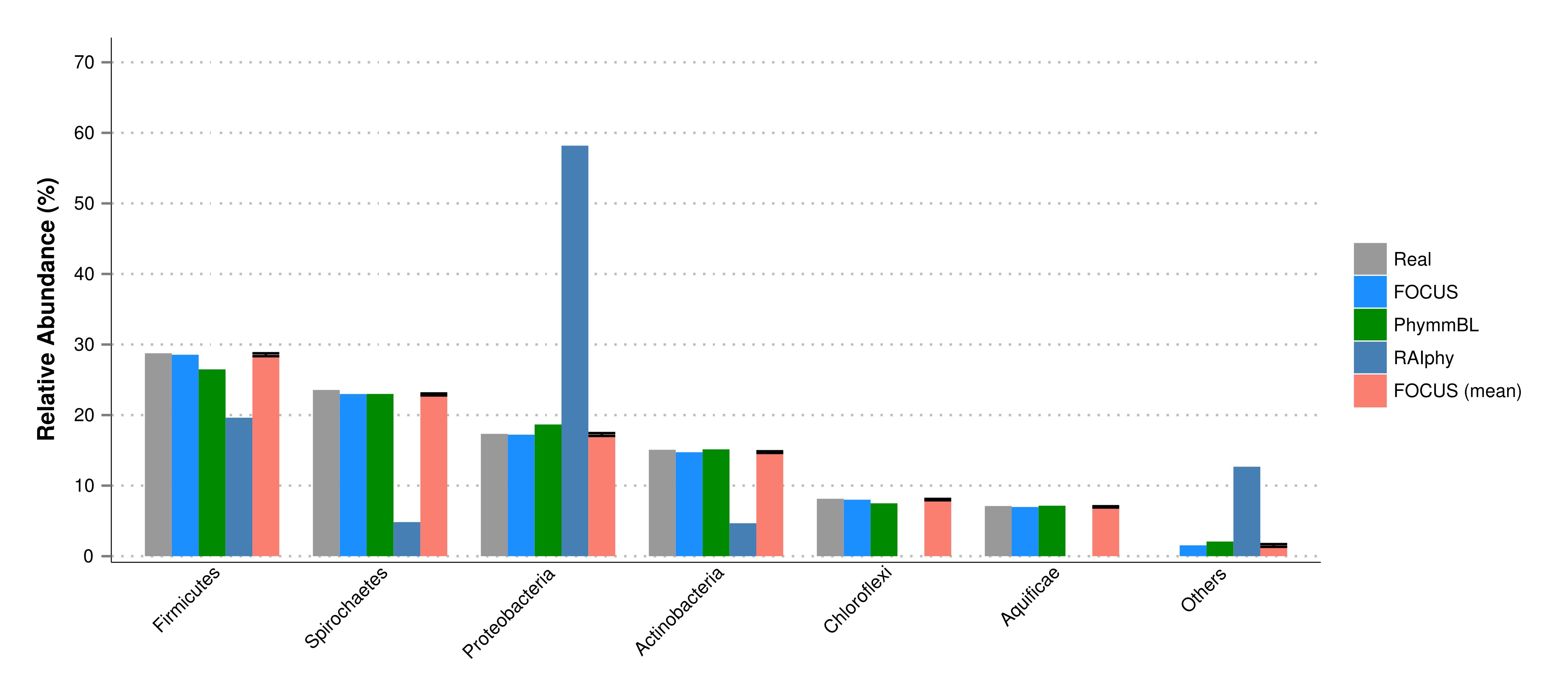
 Supplemental  Fig. 4.** Phylum-level taxonomy classification for the SimShort dataset using FOCUS, PhymnBL, RAIphy, and FOCUS (mean).


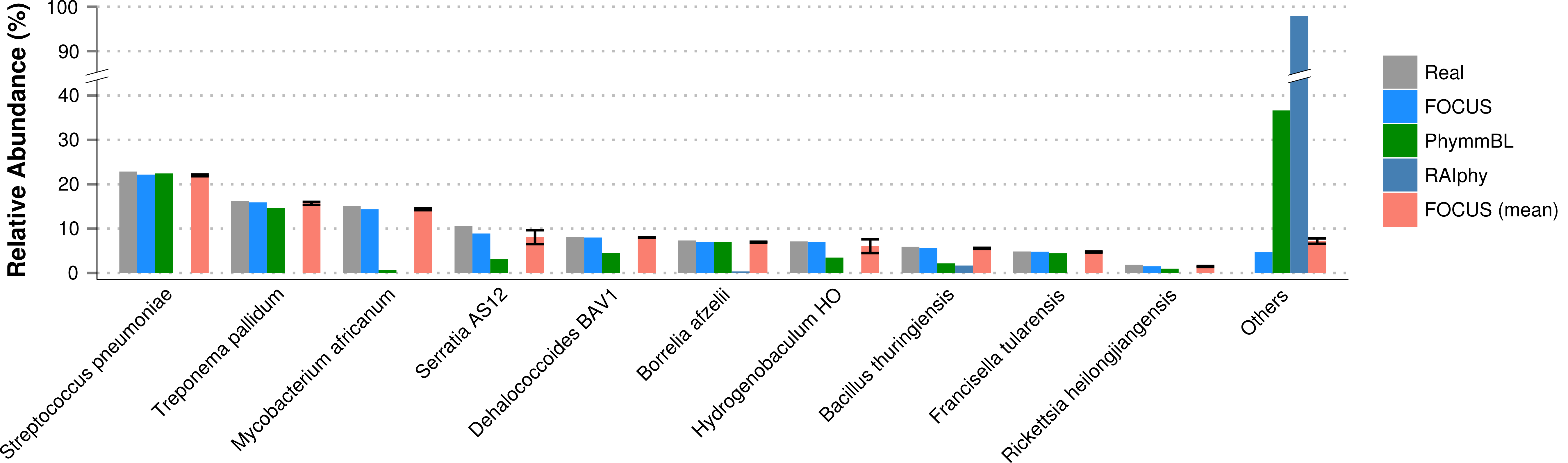


**Supplemental  Fig. 5.** Species-level taxonomy classification for the SimShort dataset using FOCUS, PhymnBL, RAIphy, and FOCUS (mean).


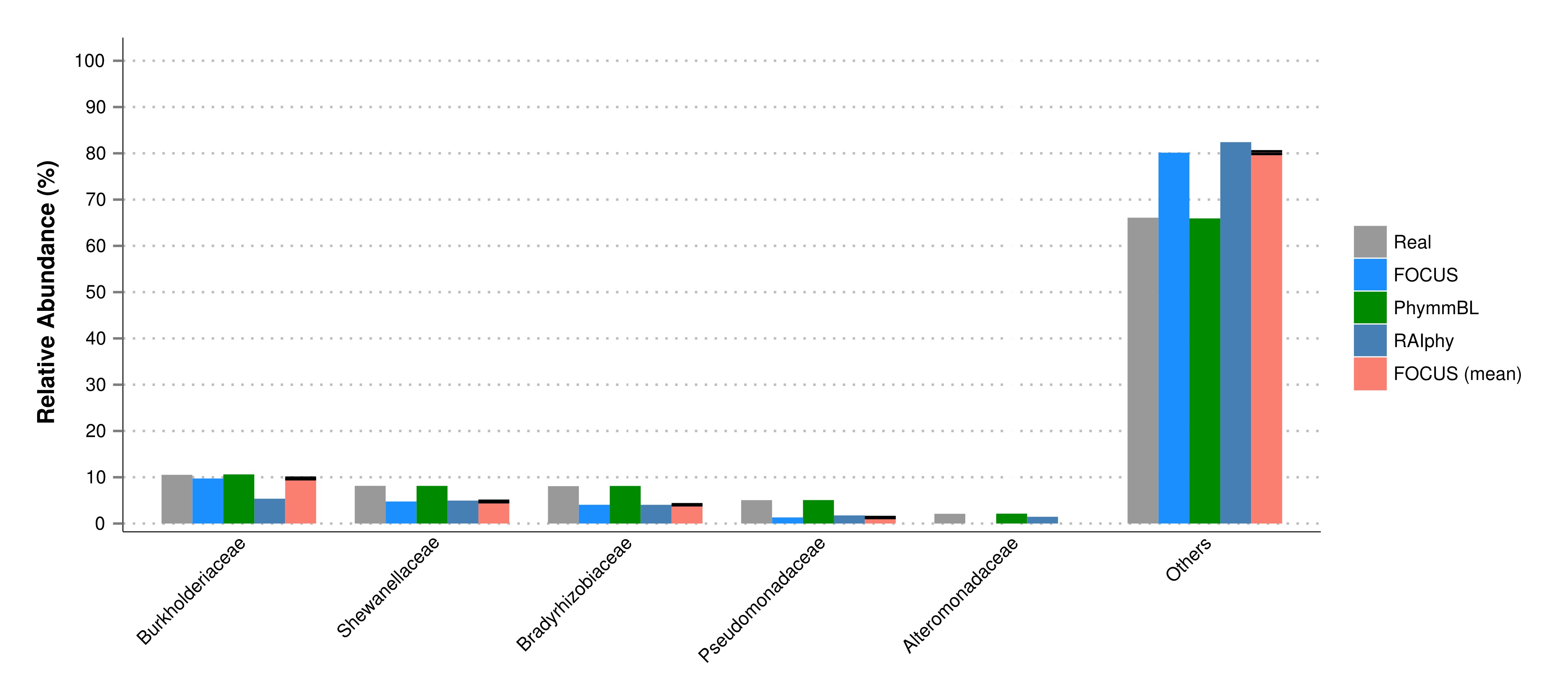


**Supplemental  Fig. 6.** Family-level taxonomy classification for the SimHC dataset using FOCUS, PhymnBL, RAIphy, and FOCUS (mean).


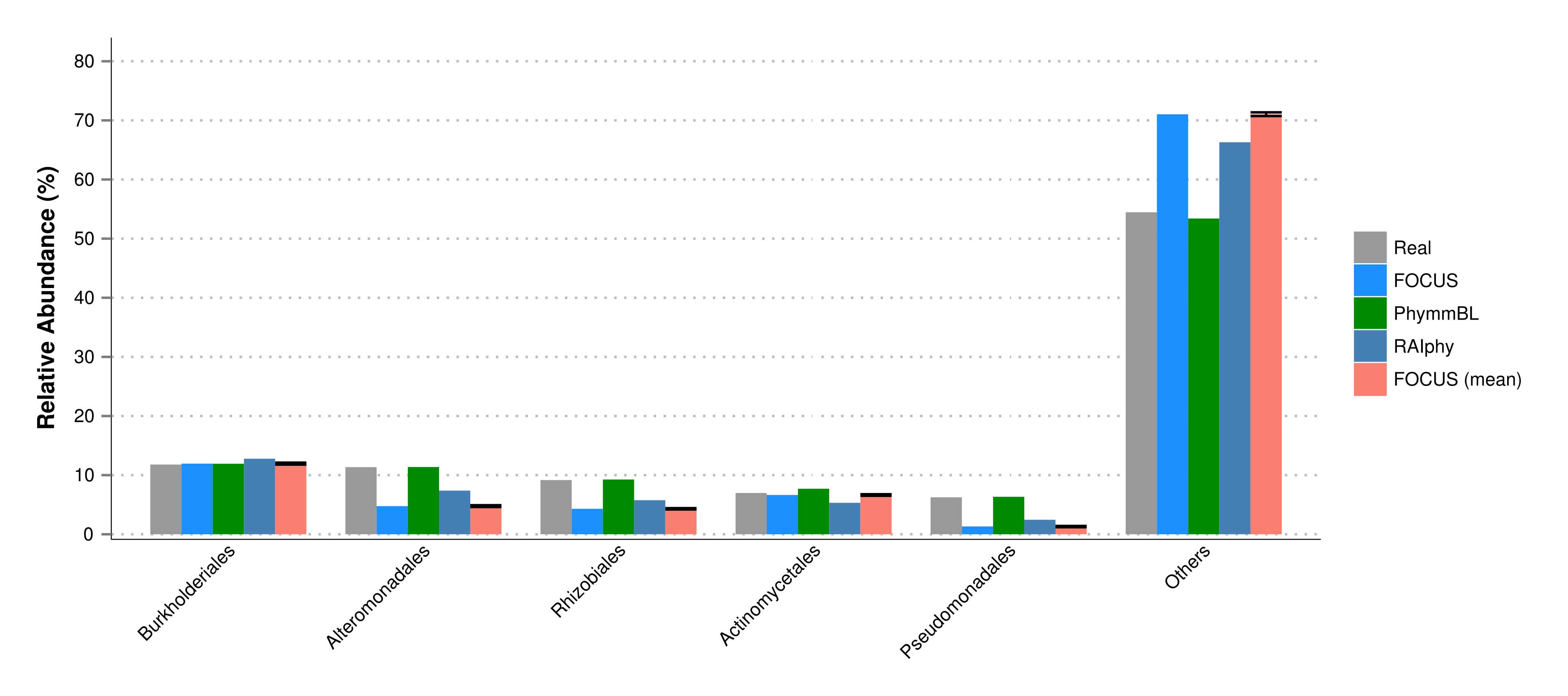


**Supplemental  Fig. 7.** Order-level taxonomy classification for the SimHC dataset using FOCUS, PhymnBL, RAIphy, and FOCUS (mean).


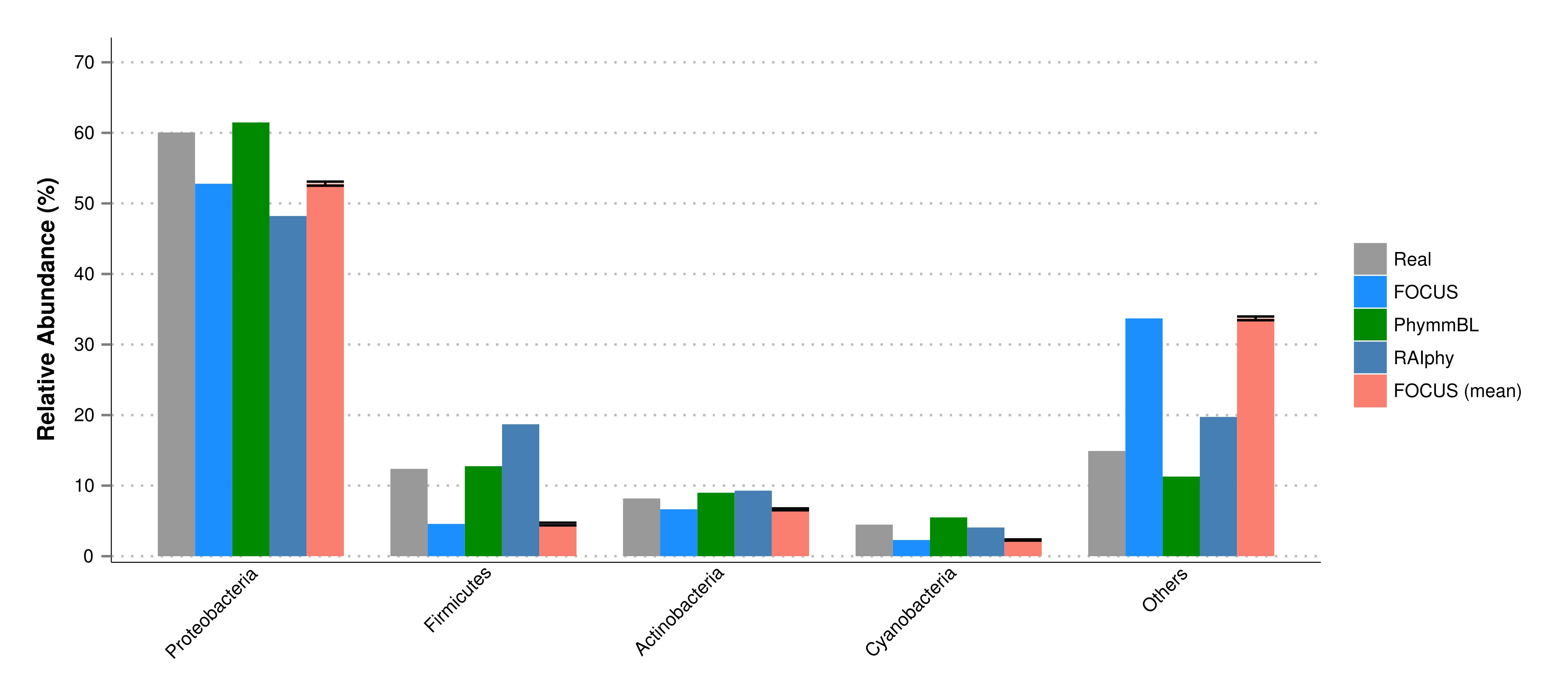


**Supplemental  Fig. 8.** Phylum-level taxonomy classification for the SimHC dataset using FOCUS, PhymnBL, RAIphy, and FOCUS (mean).
